# Statistical analysis plan for continuous positive airway pressure plus mandibular advancement therapy (PAPMAT): an adaptive randomised crossover trial comparing the benefits and costs of combining two established treatments for obstructive sleep apnoea

**DOI:** 10.1186/s13063-025-09126-9

**Published:** 2025-11-06

**Authors:** Martin Law, Yi-Da Chiu, Sofía S. Villar, Timothy Quinnell

**Affiliations:** 1 Royal Papworth Hospital NHS Foundation Trust, Cambridge, CB2 0AY UK; 2 Medical Research Council Biostatistics Unit, University of Cambridge, Cambridge, CB2 0SR UK

**Keywords:** Statistical analysis plan, Crossover, Sample size re-estimation, Adaptive design, Sleep apnoea, CPAP, Mandibular advancement device

## Abstract

**Background:**

Obstructive sleep apnoea is caused by closure of the upper airway during sleep due to excessive muscle relaxation. It is treated with continuous positive airway pressure (CPAP), a machine connected to a mask worn by a patient during sleep, which generates pressure to keep the throat open. CPAP is highly effective, but often not tolerated, sometimes due to the required pressure of the machine. Mandibular advancement devices advance the lower jaw, increasing airway space. Using such a device may open the airway enough to allow CPAP pressure to be reduced, resulting in more patients being able to tolerate using the CPAP machine.

**Methods/design:**

The PAPMAT trial is a multicentre, randomised controlled crossover trial. It will measure CPAP machine adherence for participants with obstructive sleep apnoea, comparing their adherence when using a CPAP machine alone to using a CPAP machine in conjunction with a mandibular advancement device. The sample size will be re-estimated after at least 50% of participants have completed follow-up. This document is the statistical analysis plan, which gives details of the planned analysis, including the sample size re-estimation.

**Trial registration:**

ISRCTN 33966032. Registered 18th February 2022.

**Supplementary Information:**

The online version contains supplementary material available at 10.1186/s13063-025-09126-9.

## Introduction

### Preface

This study will assess an intervention that could enhance the effectiveness of an existing NICE-recommended therapy for obstructive sleep apnoea (OSA). Both mandibular advancement devices (MAD) and continuous positive airway pressure (CPAP) therapy are part of NHS clinical services. Should the results prove effective and cost-effective, the new ‘combination therapy’ could be rapidly introduced as an additional, efficient tool to help the significant number of patients who struggle with ‘monotherapy’.

OSA is caused by closure of the upper airway during sleep due to excessive muscle relaxation. Pauses in breathing caused by total (apnoeas) or partial (hypopnoeas) airway closure cause oxygen levels to drop and also cause brief awakenings that result in excessive daytime sleepiness. OSA impacts all aspects of life, including social and work performance and driving safety, and impairs quality of life (QoL) [1]. OSA has other important health effects. It is linked to high blood pressure [2] and a 2.5 times higher risk of developing cardiovascular disease including heart attack and stroke [3]. OSA is associated with abnormal heart rhythms including atrial fibrillation, which itself undermines QoL and daytime functioning and increases stroke risk.

OSA is straightforward to diagnose and is treated with CPAP. A patient wears a mask connected to a small electric air pump that generates pressure to keep the throat open during sleep. CPAP is highly effective in treating OSA, improving daytime sleepiness, functional status, and QoL. It is the treatment of choice for moderate-to-severe OSA [4].

Unfortunately, not everyone tolerates CPAP, and adherence rates range from 20 to 83%, so untreated OSA is prevalent [5]. Uncomfortably high pressure is a possible reason for CPAP intolerance. Patients start on a low pressure, which is increased until OSA is controlled. For some patients, the required pressure is higher than they can tolerate, making CPAP difficult to sleep with and defeating the purpose of treatment. In a nationwide survey of CPAP users, we found 68% had had difficulty using CPAP due to the pressure being delivered. One method to try to improve CPAP tolerance is to use an auto-titrating CPAP machine. These aim to make CPAP more tolerable by continually monitoring for apnoeas and adjusting pressure according to whether they are occurring. Meta-analyses suggest auto-CPAP leads to a small improvement in CPAP adherence, although average pressure delivery is not much different to fixed pressure machines. While auto-titration may help some patients to tolerate CPAP, it does not address all pressure-related issues, and in some patients, the pressure needed to control OSA exceeds CPAP’s capacity, leading to ‘breakthrough’ OSA. More expensive machines deliver much higher pressures, but these are not available in every NHS service, and there is no robust evidence that they are better tolerated [5].

Mandibular advancement devices (MAD) are worn in the mouth during sleep. They treat OSA by advancing the lower jaw and increasing airway space. MAD are not as good as CPAP at treating OSA. However, they are useful in milder disease, are cost-effective [6], and sometimes help people with more severe OSA who cannot tolerate CPAP [4].

Combining MAD with CPAP could potentially open the airway enough to allow CPAP pressure to be reduced. This could allow patients requiring higher CPAP pressures to tolerate treatment better and use CPAP more, increasing the chance of improving OSA symptoms and avoiding longer-term health impacts. If this study shows combination CPAP-MAD does reduce CPAP pressures, then it would also establish the utility of this intervention for improving experiences of patients with ‘breakthrough’ OSA.

Up to 24% of middle-aged men and 9% of women have at least mild OSA (apnoea-hypopnoea index (AHI) ≥ 5/h) [7]. A total of 2% to 7% of adults have associated excessive daytime sleepiness, known as OSA ‘syndrome’ (OSAS). Untreated OSA has substantial socioeconomic costs in terms of reduced work productivity, sickness, and road traffic accidents [8]. There are also healthcare costs of conditions that may be exacerbated by OSA [9]. Elderly and middle-aged patients with OSA have roughly double the healthcare costs of age-matched controls [10] which reduce considerably when they are treated effectively [11]. However, the limited utility of other treatments for more severe OSA means that patients who cannot tolerate CPAP often remain untreated, so new ways to modify CPAP pressure delivery are still needed [12].

If the results of this study show CPAP-MAD combination therapy to be effective and cost-effective, then it would be straightforward to makfe this treatment widely available. Results of our national patient survey suggest there is a significant proportion of OSA patients around the UK who need help and could benefit. Successfully treating these patients would not only improve their symptoms but could reduce the risk of longer-term conditions associated with untreated OSA. At population level, there is strong evidence that CPAP adherence is cost-effective [13] and superior to no treatment after a minimum of 2-year treatment [14]. It has been estimated that effectively treating all OSA could save £5 million/year [15]. The British Lung Foundation estimated in 2014 that £5000/QALY could be saved if patients were effectively treated.

### Purpose of the analyses

The analyses will assess the efficacy, safety, and cost-effectiveness of the combination therapy on the outcomes of patients with moderate to severe OSA. The analysis results will be included in the study report.

## Study objectives and outcome measures

### Study objectives

The primary objective is to determine if combining MAD with CPAP therapy makes it easier for patients with moderate to severe OSA to tolerate CPAP (in comparison with the CPAP therapy only), measured through increasing adherence to treatment. Therefore, the primary outcome is testing for superiority.

**Table:** 

Objectives		Outcome measures
**Primary objective**	Does combining MAD with CPAP therapy make it easier for patients to tolerate CPAP, thereby increasing adherence to treatment?	Difference in CPAP adherence (hours per night) between treatment arms. Relevance: adherence to CPAP should aid OSA
**Secondary objectives**	Does combining MAD with CPAP therapy reduce CPAP pressure requirements?	•Difference in mean CPAP pressure between treatment arms. Relevance: high CPAP pressure is a possible reason for CPAP intolerance
	Does combination therapy objectively improve whole-night OSA control or office blood pressure compared to CPAP alone?	•Between-arm 4% ODI and AHI differences* •Diastolic and systolic blood pressure
	Bespoke measurement of patient-specific resource and health service use	•Length of telephone support between arms •Time preparing and supporting/refitting MAD/CPAP •Use of medication and diagnostic tests •Visits to secondary care and use of primary and community care
	Does combination therapy improve patient-reported outcome measures (PROMs) compared to CPAP alone?	Patient-reported outcome measures are as follows: •Epworth Sleepiness Scale score (ESS) •Functional Outcomes of Sleep Questionnaire (FOSQ)/EuroQoL •EQ-5D-5L •Short form-36 (SF-36) •Pittsburgh Sleep Quality Index (PSQI) •Patient satisfaction and treatment preference •Side effects
	Is combination MAD with CPAP therapy cost-effective compared with CPAP alone?	•EQ-5D-5L and SF-6D quality-adjusted life years •Health service use

*From WatchPAT home sleep study worn for one night within final week of each treatment arm

### Outcomes in detail

**Table:** 

Domain	Specific measurement	Analysis metric	Aggregation method	Time point
CPAP adherence	Time spent each night using CPAP. Mean value used if nightly data unobtainable	Difference between arms (within patient if mean value used)	Continuous: each measurement used in mixed model. Mean value used if nightly data unobtainable	Downloaded at baseline, start of each treatment, and at each night (after 2-week acclimatisation per treatment)
CPAP pressure	Mean pressure measured each night on CPAP machine. Mean value used if nightly data unobtainable	Difference between arms (within patient if mean value used)	Continuous: each measurement used in mixed model. Mean value used if nightly data unobtainable	Downloaded at baseline, start of each treatment, and at each night (after 2-week acclimatisation per treatment)
4% ODI	Frequency of drops in oxygen saturation by at least 4% from baseline per hour of sleep, measured using WatchPAT home sleep study	Difference between arms	Mean value per hour	One night in final week, per treatment
WatchPAT—AHI	Frequency of apnoeas and hypopnoeas per hour of sleep, measured using WatchPAT home sleep study	Difference between arms	Mean value per hour	One night in final week, per treatment
CPAP—AHI	Frequency of apnoeas and hypopnoeas per hour of sleep, acquired from download from CPAP machine	Difference between arms	Mean and Median value	Collected with CPAP downloaded at baseline and at start of treatment (visit 3) and then reported for both final 4 weeks of treatment and full 10 weeks of treatment
Blood pressure	Systolic blood pressure	Difference between arms	Continuous: single value	Visit 3 and end of each treatment period
	Diastolic blood pressure	Difference between arms	Continuous: single value	Visit 3 and end of each treatment period
Length of telephone support	Frequency of telephone support	Difference between arms	Continuous: minutes	End of each treatment period
Use of anti-hypertensive medication	Type, dose, and duration of medications used	Cost per arm for each medication type	Continuous: multiplied by unit cost and aggregated to total cost per patient	Visit 3 and end of each treatment period
Use of diagnostic tests—Watch PAT	Number of each type of diagnostic test used	Cost per arm	Continuous: multiplied by unit costs and aggregated to total cost per patient	End of each treatment period
Sleepiness	ESS score	Difference between arms	Continuous	Visit 3 and end of each treatment period
Quality of life	FOSQ	Difference between arms	Continuous: score	Visit 3 and end of each treatment period
	EuroQoL-VAS	Difference between arms	Continuous: score	Visit 3 and end of each treatment period
	EQ-5D-5L	Difference between arms	Continuous: score	Visit 3 and end of each treatment period
	SF-36 scores and SF-6D values	Difference between arms	Continuous: score	Visit3 and end of each treatment period
Sleep quality	PSQI	Difference between arms	Continuous: score	Visit 3 and end of each treatment period
Treatment preference	MAD + CPAP or CPAP	Number per arm	Binary	End of second treatment period
Ongoing treatment decision	MAD + CPAP or CPAP or other	Number per arm	Trinary	End of second treatment period
Participant reported side effects during treatment period with MAD + CPAP	Individual participant reports side effects experienced	Side effects experienced Y/N and then free text to add detail	Categorical: qualitative	End of second treatment period
Quality-adjusted life years	EQ-5D quality-adjusted life years. Utility value at a time point based on UK tariff for EQ-5D	Difference between arms	Continuous. Area under the curve calculated as quality-adjusted life years. Mean difference	Visit 3 and end of each treatment period
Quality-adjusted life years	SF-6D quality-adjusted life years (subset of SF-36). Utility value at a time point based on UK tariff for SF-6D	Difference between arms	Continuous. Area under the curve calculated as quality-adjusted life years. Mean difference	Visit 3 and end of each treatment period
Health service use	Use of GP or nurse in-person, at home, via telephone, or online, dentist, NHS111, trial helpline, ambulance, A&E, hospital outpatient, and hospital overnight admission. Average (SD) use per service type per patient	Difference between arms	Each service type valued using specific unit cost, with total cost for all services aggregated at patient level summarised as mean and SD at group level	Visit 3 (regarding last 1–12-month use and travel costs) and end of each treatment period

#### Adherence

Adherence to treatment is an important aspect of clinical trials, with such data providing context for any observed treatment effect. In this study, adherence to treatment is itself the primary endpoint: the number of hours of CPAP machine use. For each participant, this is recorded automatically each time the machine is used. While the machine is intended to be used nightly, and adherence is recorded by the CPAP machine every night, it currently remains unclear whether such data will be available for analysis at the “per night” level. If it is not, we will receive the following data for each treatment arm (both of which include CPAP use):

- Average number of hours used over full non-acclimatisation treatment period (10 weeks)
- Number of days CPAP was used (over the 10-week period)
- Number of days CPAP was used for at least 4 h (over the 10-week period)

Adherence will be presented using the above data.

Some participants will have a non-acclimatisation treatment period that is not 70 days exactly, due to the timing of visit 5. If the period is greater than 70 days, data will be used from their most recent 70 days only. If the period is less than 70 days, all non-acclimatisation treatment data will be used. In both cases, the participant’s non-standard treatment period will be recorded. If nightly adherence data are available, then these will be presented, possibly as small multiples, one for each participant.

## Study methods

### General study design and plan

The study is a two-centre, adaptive 2 × 2 randomised crossover design, comparing the adherence of CPAP + MAD (combination therapy) to CPAP in patients with moderate to severe OSA. Patients will be recruited from May 2022 until the re-estimated sample size is reached, which is expected to be late 2025 (as of April 2025). Events are scheduled as Table 1.

**Table 1:** Schedule of events

	**V1**	**V2**	**Randomisation**	**V3/*research visit 1***	**V4/*research visit 2***		**V5/*research visit 3***	
				**CPAP**1^st^	**CPAP**1^st^	**CPAP-MAD 1**^st^	**CPAP**1^st^	CPAP-MAD 1^st^
**Time interval of visit (weeks)**	1	6–7	8–10	9–10	19–20	21–22	31–32	31–32
**Introduction to study and PIS**	X							
**consent**		X						
**Medical history**		X						
**Physical exam/dental check**		X						
**Clinical review for CPAP pressure**		X						
**CRF completion**		X			X	X	X	X
**Study questionnaires**				X	X	X	X	X
**MAD moulding**		X						
**MAD quality check**			X					
**Randomisation**				X				
**MAD/CPAP fitting**				X				
**Switch auto-CPAP**				X				
**Begin study intervention CPAP or CPAP + MAD**				X	X	X		
**CPAP data download**					X	X	X	X
**Overnight sleep study**					X	X	X	X
**Return of sleep diary**						X	X	
**Clinical review and treatment preference**							X	X
**Completion of service use questionnaire**							X	X
Adverse & serious adverse events assessed				X	X	X	X	X

### Inclusion–exclusion criteria and general study population

#### Inclusion criteria

- Adults with moderate to severe OSAS defined by a 4% oxygen desaturation index (4%ODI) or apnoea hypopnoea index (AHI) ≥ 15/h
- Symptomatic daytime sleepiness (Epworth Sleepiness Scale (ESS) score ≥ 9
- Auto-titrated CPAP pressure ≥ 14 cm water

#### Exclusion criteria

- Inadequate dentition or other contraindication to MAD determined by clinician or trained CPAP provider
- Co-morbid sleep disorder that might affect the patient’s ability to comply with treatment or benefit from therapy or confound the interpretation of results
- Unstable cardiorespiratory disease or other disorder/factor judged by the clinician to preclude trial participation due to safety concerns or significant potential to confound interpretation of results.
- Previous MAD or CPAP use (predating current treatment)
- Other reason for inability to comply with trial protocol

### Randomisation and blinding

Randomisation will be undertaken by using permuted block randomisation with random permuted block sizes of 4 and 8. The allocation ratio of the combination therapy to CPAP is 1:1. The randomisation service will be hosted by the sealed envelope. There will be no stratification.

Due to the nature of the intervention, patients and clinical staff cannot be blinded whilst the patient is receiving randomised therapy. However, a team of research staff will collect data on outcomes, and these staff will be blinded. The interim analysis will be done by an independent unblinded statistician so that the trial statisticians can remain blinded until the final analysis.

## Sample size

### Initial target sample size

A sample size of 64 patients was selected based on power (90%) and two-sided type-I error (5%) considerations for the primary endpoint of average hours of CPAP usage within the treatment window of 10 weeks and a drop-out rate of 10%.

Using TOMADO [4] data, we estimated a pooled variance of differences in average treatment use (in hours per night) between each type of MAD (three types in total) and no treatment. The pooled variance was obtained using the equation as follows:

$${\widehat{\upsigma }}_{m}^{2}= \sum_{i \ne j}^{4}{s}_{ij}^{2}({n}_{ij} - 1) /\sum_{i \ne j}^{4}({n}_{ij} - 1) ,$$

where ${\widehat{\sigma }}_{m}^{2}$ is an estimate of the variance of the treatment difference, ${n}_{ij}$ is the number of non-missing pairs of responses for treatments $i$ and $j$, and ${s}_{ij}$ is the sample standard deviation of the vector of paired differences for treatments $i$ and $j$. The pooled variance estimate obtained was ${\widehat{\upsigma }}_{m}^{2} = 5.51$. Using this estimate as a reference (making the assumption that patient variability between CPAP and CPAP + MAD will be similar), we determine that a sample size of 58 patients is sufficient to detect a 1-h difference in the average use of the between the two treatments, with 90% power and two-sided 5% type-I error rate. There is a single primary outcome, and so there is no need to adjust for multiplicity. To allow for estimated loss to follow-up (informed from TOMADO experience), we intend to randomise an additional 10% of patients to give a final sample size of 64.

The associated formula for calculating the sample size above is from Eq. (9) in the guideline paper [16]:

$$\text{n}={\left({\text{Z}}^{-1}\left(1-{}^{{\upalpha }}\!\left/ \!{}_{2}\right.\right) + {\text{Z}}^{-1}\left(1-\upbeta \right)\right)}^{2}\frac{{\widehat{\sigma }}_{m}^{2}}{2{\varepsilon }_{R}^{2}}$$

Here, ${\varepsilon }_{R}$ is the mean difference between adherence in the two arms, under the alternative hypothesis. The result is the *per treatment* sample size.

### Adjusted target sample size

The assumptions used for the initial sample size calculation were based on the data of the TOMADO trial. As this trial (PAPMAT) uses a different therapy comparison to the TOMADO trial, we found that the sample size calculation could be sensitive to the standard deviation of the difference in primary endpoint between two arms. PAPMAT has been designed as an adaptive trial with an interim sample size re-estimation planned after at least 29 patients complete the data collection for the primary outcome at the end of two 10-week treatment windows.

At the interim analysis, we will use the data accumulated so far to re-estimate the required sample size. Treatment efficacy will not be assessed at the interim analysis. After the interim analysis, the sample size of the trial will be updated with a maximum sample size of up to 90 patients. There are three possible outcomes from the sample size re-estimation, summarised in Table 2.

**Table 2:** Recommended sample size from interim sample size re-estimation and course of action

Recommended sample size from interim sample size re-estimation	Course of action
≤ 64	Continue recruitment to 64
64–90	Continue recruitment to the new recommended sample size
> 90	Continue recruitment to 90

The sample size re-estimation will be done by an independent statistician or a delegate to allow the trial statisticians to remain blinded, in order to preserve the type-I error rate. This sample size adaptation may prevent an underpowered trial if moderate deviations from the assumptions made for the initial sample size calculation are observed. Note that the maximum recruited number above (90 patients) was estimated based on the enrolment information from the TOMADO trial (4–5 patients per month) and a possible maximum extension period (6 months) and so is a pragmatic rather than statistical choice. The figure is subject to change according to recruitment data and other factors during the interim analysis stage.

Any recommended adjustment to the sample size resulting from the pre-specified interim analysis will be formally documented as an amendment to both the clinical study protocol and the statistical analysis pPlan (SAP). These updated documents will clearly record the revised sample size and the justification for its modification, ensuring full transparency and adherence to the protocol requirements.

## General considerations

### Timing of analyses

#### Interim analysis

The proposed interim analysis—with the purpose to perform a sample size re-estimation—will be conducted when at least 29 patients have completed the primary endpoint, which is initially expected to happen at month 21. The primary rationale behind the timing of the interim analysis is based on the minimum sample size needed to make precise enough estimates of the variance parameters in each arm of the trial. A range of published literature exists on the topic of minimum sample size for pilot studies, which is akin to the sample size requirements for an interim sample size re-estimation or internal pilot.

Traditionally, a “rule of thumb” approach was used to set the sample size for pilot studies at around 30, though there have also been a number of important papers published giving a more scientific basis for the selection of sample size for pilot studies.

For two-arm studies with a continuous outcome, Julious (2005) recommends a total sample size of 24 [17] and Keiser (1996) recommends a total sample size of 20–40 [18], whilst Sim (2011) recommends a total sample size of at least 50 [19] and Teare (2014) recommends a total sample size of at least 70 [20]. More discussions can be found in the work of Whitehead et al. [21].

Our recommendation for performing the interim sample size re-estimation after at least 29 patients have completed their primary endpoint sits towards the lower end of these figures. This must be carefully balanced with the duration of treatment and follow-up, particularly in the case of trials using a cross-over design, which will naturally delay the point at which the interim analysis occurs. In addition, if there is a large amount of missing data for the primary endpoint, then the interim analysis may be delayed.

#### Final analysis

The final analysis will be undertaken after data cleaning and following database lock, which may take place once the final participant has completed their 24 weeks of total follow-up.

### Trial population

#### Recruitment and eligibility

The progress of all participants through the trial will be shown within a CONSORT flow diagram. This will include (but is not limited to) the following:

- Assessed for eligibility at screening
- Eligible at screening
- Ineligible at screening*
- Eligible and randomised
- Eligible but not randomised*
- Received the randomised allocation
- Did not receive the randomised allocation*
- Lost to follow-up*
- Discontinued the intervention*
- Randomised and included in the primary analysis
- Randomised and excluded from the primary analysis*

*Reasons will be provided.

Eligibility criteria are provided in the protocol. The frequency of each exclusion criterion will be reported.

#### Withdrawal and follow-up

The level of consent withdrawal will be tabulated. Timing of withdrawal and loss to follow-up data will be incorporated into the CONSORT diagram. Reasons for withdrawal and loss to follow-up will be tabulated, where known.

#### Baseline participant characteristics

Participant characteristics at baseline will be reported for both the analysis population and for all randomised participants. Categorical data will be summarised by numbers and percentages. Continuous data will be summarised by mean, SD, median, IQR, and range. No statistical analysis of baseline data is planned. Characteristics include (but are not limited to) standard demographic information such as age, sex, and BMI, as well as those listed in “Outcomes in detail” above.

### Analysis populations

Before database lock, each patient will be included or excluded from each of the analysis populations defined below. This will be carried out prior to unblinding to avoid bias of any analyses.

Both statistics and health economics teams will receive the same data sets for final analyses.

#### Safety population

The safety population includes all subjects entered into the study, regardless of whether they received any study treatment. The safety population will be used to provide summary statistics on adverse events and serious adverse events.

The interim analysis will include safety data. In this instance, the safety population should include all subjects entered into the study at the time of database lock for the interim analysis, regardless of whether they are formally included in the interim analysis population. We will include anyone who has withdrawn if they consent to their data still being used. Safety data will be split into participants who are still in the trial and those who are not.

#### Interim analysis population

The interim analysis population will include all patients who have completed the study, with the exception of those who provide very little adherence data (see section “Missing data at interim analysis”).

#### Intention-to-treat population

The intention-to-treat analysis population includes all subjects who were randomised, regardless of whether they received the treatment randomly allocated to them and also completed the two therapies (CPAP or CPAP-MAD combination). The data will be analysed assuming that the patient received both treatments and in the order that they were randomly allocated.

#### Per-protocol population

In general, the per-protocol population is the group of participants who are most compliant with the protocol and/or “received the randomised treatment” for some definition that is pre-specified and appropriate for each study. However, it is not possible to accurately assess the extent to which participants receive the CPAP + MAD combination treatment, as days of combined use are not recorded. As such, no per-protocol population is defined, and no per-protocol analysis is planned.

#### Subgroup-analysis population

The planned subgroup-analysis population is comprised of the participants from the two recruiting centres (Royal Papworth Hospital and Bristol Royal Infirmary).

### Missing data

Missing data will be quantified per variable with missing frequencies and proportions (%) by groups. All essential variables for the outcomes are expected to be complete or at least low missing percentages (less than 5%) before starting analysis. Variables with > 25% missing data may not be used in modelling as covariates or outcomes unless they have clinical importance. Such variables will be summarised and reported if listed as intended covariates or outcomes.

For participants who discontinue the allocated treatment CPAP + MAD but continue to use the CPAP machine, we will use their adherence data as if they were still continuing their allocated treatment (i.e. intention to treat). For participants who discontinue the allocated treatment CPAP (and thus no longer provide adherence data), we will impute their adherence as 0 h for each remaining day. This pragmatic approach reflects the primary endpoint, which is the number of hours of CPAP adherence, and the wider research question, which is to examine if CPAP adherence is greater for participants allocated to CPAP + MAD compared to those allocated to CPAP only.

For the primary endpoints and the planned covariates for the primary analysis, if the proportion of missing data is at most 5% or the mechanism is missing completely at random (MCAR), we will ignore the missing data and run a complete-case analysis. If the proportion of missing data is greater than 5% and not MCAR, we may impute the missing data by using multiple imputation by chained equations techniques (MICE) [22]. The imputed data would be analysed as a sensitivity analysis for the primary analysis only. When MICE is used, the imputation model will include endpoints and predictors of baseline variables with missing data. The number of imputed datasets depends on the overall missing proportion in the analysis data [23]. The imputation methods for variables with missing values may depend on the variable type or the fixed formula (such as BMI). Sensitivity analysis will be carried out to evaluate the robustness of the estimated coefficients where missing data has been imputed. Missingness indicators will be created for examining the mechanism by using statistical testing (such as Little’s test [24], *t*-test, and chi-squared test) or graphical inspection.

#### Missing data at interim analysis

Participants who provide at least 14 days of CPAP adherence data for both treatment arms will be included in the sample size re-estimation; otherwise, they will be excluded. If > 10% of participants do not reach this threshold, we will attempt to include their data, appropriately weighted in comparison to the more complete data from other participants.

## Summary of study data

### Data checks

Outliers in continuous variables will be detected by examining ranges and plotting distributions by treatment group. Categorical variables will be tabulated by treatment group, and unexpected distributions or data points will be further checked. Consistency checks between two or more variables will also be performed, e.g. plotting weight against age. Clear outliers, e.g. > 4 standard deviations from the centre of distribution, will be removed and regarded as a missing value, and their effect will be investigated through sensitivity analyses. However, a series of potential outliers occurring in a skewed distribution may be considered to hold, and in these cases, the data will be transformed by nonlinear function (such as log or squared root).

### Descriptive statistics

All variables will be summarised using the following descriptive statistics: for continuous variables, the non-missing sample size, mean, standard deviation, median, 1 st and 3rd quartile (represented as an interval), maximum and minimum, and missing percentages, and for categorical variables, the frequency and percentages (based on the non-missing sample size) of observed levels will be reported. 

### Derived variables

#### EQ-5D-5L

The mapping utility value of EQ-5D-5L will be created through crosswalk index value sets [25] or new EQ-5D-5L value sets should these become available. The value ranges from −0.591 to 1.

#### BMI

Body mass index (BMI) will be calculated using the following formula:

$$BMI=\frac{m}{{h}^{2}}$$

where *m* = mass (in kilograms)

*h* = height (in metres)

#### SF-36

SF-36 health state responses were converted to the Short Form questionnaire-6 Dimensions (SF-6D) utility scale using values from the UK population [26]. The converted single index value ranges from 0 to 1.

### Protocol deviations

We do not expect protocol deviations that could impact the analysis. However, if such an event happens, we will amend this SAP for specifying the methods used to describe and analyse them if necessary. One possible protocol deviation is that a participant beginning on the CPAP + MAD treatment then routinely uses their MAD after they have switched to the CPAP-only treatment. This may manifest in a smaller treatment effect in participants who begin on the CPAP + MAD treatment compared to participants who begin on the CPAP-only treatment. This difference will be reported, in addition to any direct admission of such deviations recorded in the CRFs.

If a protocol deviation occurs due to an eligibility failure that is discovered in the latter stage, the associated patients will be excluded from the primary and secondary analysis populations. The sample size may also be inflated depending on the granularity of such issues found at the interim analysis.

## Analysis

### Interim analysis

Sample size re-estimation is the primary purpose of the interim analysis. The trial statistician will prepare code to calculate the variance of the difference in mean adherence (in hours) between the CPAP + MAD combination therapy and the CPAP therapy, with the treatment group labelled using a dummy/scrambled randomisation list. An independent statistician will then run the code using the real randomisation list provided by the data manager and produce updated estimates of the variance for the crossover difference. The original sample size calculation will be repeated with the updated estimate to provide an updated sample size estimate, which will be reported to the DMEC. The equation used for both sample size calculation and the interim analysis sample size re-estimation was provided by Dr. Yi-Da Chiu.

The frequencies of the AEs and SAEs will be reported. Note that AEs that are OSA symptoms will not be reported.

In addition, information about patient recruitment and compliance (in terms of hours of CPAP use per am and number of days of CPAP use) will be reported at the interim analysis. The summary of recruitment data will be presented as well as treatment group and month where appropriate. As the primary endpoint is adherence to treatment, a standard approach to reporting compliance may jeopardise blinding. As such, the reporting of compliance data will be limited.

The trial statisticians and data management team will assess data quality to ensure collecting high-quality data.

Treatment efficacy will not be assessed at the interim analysis. In the event that the DMEC requests additional data analyses at the interim stage, the trial statistician will be responsible for providing these (via the independent statistician if unblinding is required). There is no planned adjustment of the significance level. The trial will only be stopped early on the advice of the DMEC; there are no pre-planned stopping guidelines related to treatment efficacy.

### Primary analysis

The primary outcome is the difference in CPAP adherence between treatment arms.

We will use a mixed-effects model to fit daily CPAP adherence as a function of oxygen desaturation, ESS at baseline, age, gender, BMI, and site as fixed effects and random intercept for each participant to account for variation in adherence within participants. Our primary research question corresponds to a test of the effect of treatment, represented by ${\beta }_{T}$ in the model below. The assumptions we make, and how they would be tested, are as follows:

- Errors are independent and have constant variance, and linear relationship between adherence (response) and covariates is as follows:
  o Plot of residuals against fitted values
- Errors are normally distributed as follows:
  o QQ plot of residuals

If the assumptions are found to not hold, appropriate transformations will be made to the data.

If it is not possible to obtain adherence per night and instead we can only obtain mean adherence for each participant per treatment, the above analysis will not be possible. Instead, we will use a linear regression, subtracting the mean adherence on the control arm (CPAP) from that in the experimental treatment arm (CPAP + MAD), for each participant, adjusting for the same covariates as specified immediately above. In either case, we will report the 95% confidence interval for the difference in adherence between the two arms. If the lower bound of the 95% confidence interval is greater than 1 (hour), we will conclude that there is evidence of increased CPAP adherence on the experimental (CPAP + MAD) arm compared to the standard (CPAP) arm. The assumptions tested will remain the same. The form of the mixed-effects model is as follows.

$${y}_{ijk} = {\beta }_{0}+{{\beta }_{T}}{T}_{j}+{\sum }_{p=1}^{P}{\beta }_{p}{x}_{pi}+{\gamma }_{i}+{\varepsilon }_{ijk}$$

$${\gamma }_{i}\sim N\left(\left(0, {\tau }^{2}\right)\right), {\varepsilon }_{ij}\sim N\left(\left(0, {\sigma }^{2}\right)\right),$$

where

$$i=1, 2, \dots , N; j=0, 1; k=1, 2, \dots {n}_{i}$$

Note that this formula was not used for the original sample size calculation, nor will it be used for the sample size re-estimation. This is due to data availability considerations: this analysis requires per-day adherence data for each participant, whereas the sample size calculation and re-estimation formula require only mean CPAP adherence for each participant.

The statistical analysis will be reported according to CONSORT extension guidelines for adaptive trials [27]. In cases of missing data, the missing data mechanism will be explored, and multiple imputation may be applied as a sensitivity analysis as appropriate (details in the “Missing data” section).

### Secondary analyses

Secondary endpoints will primarily be summarised per treatment or change from baseline per treatment, with the between-arm differences reported in addition. For the difference in CPAP pressure and CPAP AHI, which is expected to consist of nightly data, the analysis will use a mixed-effects model as the primary analysis.

In addition to the patient-reported measures, the study team will initially generate the difference in the summary score (for ESS, SF-36, and PSQI) or the utility value (for EQ5D or SF-36) between baseline and each period end and conduct similar secondary analyses above. Statisticians will also report the respective between-arm differences. The same procedure as for blood pressure analysis will be performed if the data is available.

For secondary outcomes measured only once per treatment arm (or only a mean or total is reported per participant per treatment arm), the between-arm difference will be analysed using linear regression including the same covariates as the primary analysis. The estimate and 95% confidence interval for the between-arm difference will be reported for sensible values of the covariates where possible. Such outcomes include the following:

- 4% ODI
- WatchPAT — AHI
- Blood pressure
- Length of telephone support
- ESS score
- FOSQ
- EuroQoL-VAS
- EQ-5D-5L
- PSQI
- EQ-5D quality-adjusted life years (area under curve)
- SF-6D quality-adjusted life years (area under curve)

Treatment preference and ongoing treatment decisions will both be analysed as comparisons of proportions.

Cost-based outcomes will be analysed by health economics. Such outcomes include the following:

- Use of antihypertensive medication
- Use of diagnostic tests — Watch PAT
- Health service use

Side effects will be summarised per arm.

A review statistician will independently reproduce the final primary analysis and also the secondary analyses of the following:

- CPAP pressure
- 4% ODI
- AHI
- Blood pressure

### Sensitivity analyses

If daily CPAP adherence data is not available and only mean CPAP adherence is available, we will recalculate mean CPAP adherence using “number of days of CPAP use” as the denominator and also using “number of days of > 4 h’ CPAP use” Additionally, there may be a missing data analysis using multiple imputation (see the “Missing data”). The aim of these sensitivity analyses is to examine estimate robustness.

### Subgroup analyses

As the auto-titrating CPAP machines used in the two centres are different, it is necessary to investigate the inter-centre difference in the primary endpoints. The subgroup analyses will be performed as the primary analysis for each centre separately.

## Safety analyses

All safety analyses will be performed on the safety population. Adverse events (AEs) and serious adverse events (SAEs) will be summarised separately, by frequency, in terms of severity and expectedness. Each figure will have a title and legend explaining any abbreviations and figure-specific detail. Scales will be clearly labelled and kept the same across plots when appropriate.

## Reporting conventions

*P*-values ≥ 0.001 will be reported to three decimal places; *p*-values less than 0.001 will be reported as ‘< 0.001’. The mean, standard deviation, and any other statistics other than quantiles will be reported to one decimal place greater than the original data. Quantiles, such as median or minimum and maximum, will use the same number of decimal places as the original data. Estimated parameters, not on the same scale as raw observations (e.g. regression coefficients), will be reported to three significant figures.

## Technical details

This SAP has been based on the version 5.0 of the study protocol. It will be shared with the TSC and DMEC for comments and comments. If changes are consequently required, these will be made as an update.

The statistical software R will be used for the statistical analyses (www.r-project.org).

Data and analysis files will be saved to a secure, backed-up network drive and will be transferred to the location the protocol specified at the end of the trial.

## Summary of changes to the protocol

If there are any changes to the protocol that impact the statistical or health economic analyses, this SAP will be updated and version-tracked accordingly.

## Supplementary Information

Supplementary Material 1: SPIRIT checklist.

## Data Availability

As protocol, the principal investigator will have access to the final dataset. The trial statisticians will have access to a blinded full dataset for analysis, whilst the health economists will have access to an unblinded full dataset for analysis. Upon request, we will provide access to a pseudo-anonymised final dataset. We aim to increase the availability of the data if possible. The code used to analyse the data will be publicly available on the Papworth Trials Unit Collaboration GitHub page (https://github.com/ptuc-stats) and on the Open Science Framework (https://osf.io).

## Abbreviations

AHI: Apnoea-hypopnoea index
CI: Confidence interval
CPAP: Continuous positive airway pressure
CRF: Case report form
CTC: Clinical trial coordinator
DMEC: Data Monitoring and Ethics Committee
ESS: Epworth Sleepiness Score
EQ-5D: EuroQoL-5D
FOSQ: Functional Outcomes of Sleep Questionnaire
GCP: Good clinical practice
MAD: Mandibular advancement device
MAR: Missing at random
MCAR: Missing completely at random
MCID: Minimal clinically important difference
MRC: Medical Research Council
MSLT: Multiple sleep latency test
NICE: National Institute for Health and Care Excellence
NIHR: National Institute of Health Research
NRES: National Research Ethics Service
ODI: Oxygen desaturation index
OSA: Obstructive sleep apnoea
OSAS: Obstructive sleep apnoea syndrome
PIL: Participant/patient information leaflet
PPI: Patient and public involvement
PRA: Patient Research Ambassador
PSQI: Pittsburgh Sleep Quality Index
PTUC: Papworth Trials Unit Collaboration
QALYs: Quality-adjusted life years
QoL: Quality of life
R&D: NHS Trust R&D Department
REC: Research ethics committee
RSSC: Respiratory Support and Sleep Centre
SATA: Sleep Apnoea Trust Association
SF-36: Short Form-36
SF-6D: Short Form-6 dimension
TSC: Trial Steering Committee
